# The role of autoantibodies in bridging obesity, aging, and immunosenescence

**DOI:** 10.1186/s12979-024-00489-2

**Published:** 2024-11-30

**Authors:** Taylor R. Valentino, Nan Chen, Priya Makhijani, Saad Khan, Shawn Winer, Xavier S. Revelo, Daniel A. Winer

**Affiliations:** 1 Buck Institute for Research on Aging, 8001 Redwood Boulevard, Novato, CA 94945 USA; 2 Department of Laboratory Medicine and Pathobiology, University of Toronto, Toronto, ON M5S 1A8 Canada; 3 Division of Cellular & Molecular Biology, Diabetes Research Group, Toronto General Hospital Research Institute (TGHRI), University Health Network, Toronto, ON M5G 1L7 Canada; 4 Banting and Best Diabetes Centre, University of Toronto, Toronto, ON M5G 2C4 Canada; 5 Pathology and Laboratory Medicine, Mount Sinai Hospital, Toronto, ON Canada; 6 Department of Integrative Biology & Physiology, University of Minnesota, Minneapolis, MN 55455 USA; 7 Institute for the Biology of Aging and Metabolism, University of Minnesota, Minneapolis, MN 55455 USA; 8 Department of Immunology, University of Toronto, Toronto, ON M5S 1A8 Canada; 9 Leonard Davis School of Gerontology, University of Southern California, Los Angeles, CA 0089 USA

**Keywords:** Autoantibodies, Obesity, Aging, Immunosenescence, Chronic inflammation

## Abstract

Antibodies are essential to immune homeostasis due to their roles in neutralizing pathogenic agents. However, failures in central and peripheral checkpoints that eliminate autoreactive B cells can undermine self-tolerance and generate autoantibodies that mistakenly target self-antigens, leading to inflammation and autoimmune diseases. While autoantibodies are well-studied in autoimmune and in some communicable diseases, their roles in chronic conditions, such as obesity and aging, are less understood. Obesity and aging share similar aspects of immune dysfunction, such as diminished humoral responses and heightened chronic inflammation, which can disrupt immune tolerance and foster autoantigen production, thus giving rise to autoreactive B cells and autoantibodies. In return, these events may also contribute to the pathophysiology of obesity and aging, to the associated autoimmune disorders linked to these conditions, and to the development of immunosenescence, an age-related decline in immune function that heightens vulnerability to infections, chronic diseases, and loss of self-tolerance. Furthermore, the cumulative exposure to antigens and cellular debris during obesity and aging perpetuates pro-inflammatory pathways, linking immunosenescence with other aging hallmarks, such as proteostasis loss and mitochondrial dysfunction. This review examines the mechanisms driving autoantibody generation during obesity and aging and discusses key putative antigenic targets across these conditions. We also explore the therapeutic potential of emerging approaches, such as CAR-T/CAAR-T therapies, vaccines, and BiTEs, to tackle autoimmune-related conditions in aging and obesity.

## Introduction

Antibodies are essential components of the humoral immune response that neutralize pathogenic agents. However, approximately 5–8% of people develop autoimmune diseases in which autoantibodies (autoAbs) [1] bind to self-antigens, aggravating inflammation and disease progression [2]. While autoAbs have traditionally been associated with autoimmune diseases, recent findings are identifying increased levels of autoAbs across many different conditions, such as obesity and aging, not traditionally characterized as autoimmune conditions [3, 4]. Specifically, obesity and aging are commonly associated with systemic chronic inflammation and functional decline of the adaptive immune system [5, 6] which is reflected as attenuated humoral responses and amplified autoimmunity [7]. In addition, age-related declines in humoral immunity appear to be exacerbated by obesity [8], and obesity has been shown to accelerate an aging immune phenotype [9–11]. Thus, obesity and aging are risk factors for the development of chronic diseases [12], in which disease-associated autoAbs may play a role.

In fact, each individual may harbor a set of specific autoAbs, known as the autoAb reactome, capable of contributing to a wide variety of phenotypic traits [13]. The autoAb reactome has been shown to play important roles in the host response to viral pathogens [14], obesity [15], aging [16], cancer [17], and neurological disease [18]. In non-communicable diseases, such as obesity, increased levels of autoAbs in the serum are seen across a diverse array of antigens, ranging from lipids like malondialdehyde (MDA) to proteins like glial fibrillary acid protein (GFAP), to nucleic acids like double-stranded (ds)DNA [3, 19]. While autoAbs are generally associated with adverse outcomes in most conditions, autoAbs have been observed to be protective in cancer [20] and may also reduce infectious and non-infectious diseases and inflammation as cytokine-neutralizing antibodies [21, 22]. Aging is also associated with increased levels of certain autoAbs, such as antinuclear antibodies (ANA), which may predispose older individuals to higher risks of type 2 diabetes (T2D) and other age-related diseases (ARDs) [23]. Finally, healthy individuals share a common set of autoAbs that may play protective roles, such as helping B cell development and self-antigen recognition [24]. Thus, autoAb responses are highly variable among individuals or disease etiology and they can have both pathological and protective roles.

In this review, we summarize current knowledge regarding mechanisms underlying autoAb development, how autoAb production is exacerbated by aging and obesity, what key autoantigens are targeted in various tissues, and how these processes could represent future therapies in complications of obesity and aging.

## Development of autoAbs

During B cell development, B cells undergo systematic rounds of expansion, selection, and differentiation to generate antibody-secreting cells (ASCs) that ultimately defend the host against pathogenic agents. However, despite existing checkpoints that prevent autoimmunity, autoreactive B cells that produce autoAbs can emerge because of altered selection processes [25]. Most of our understanding of autoAb development comes from studies in autoimmune diseases. However, the role of aging and obesity in autoAb production has been less explored. Here, we describe the mechanisms involved in the formation of autoAbs, with a focus on how the chronic inflammation commonly associated with aging and obesity facilitates the breaching of central and peripheral immune tolerance.

**Fig. 1:**
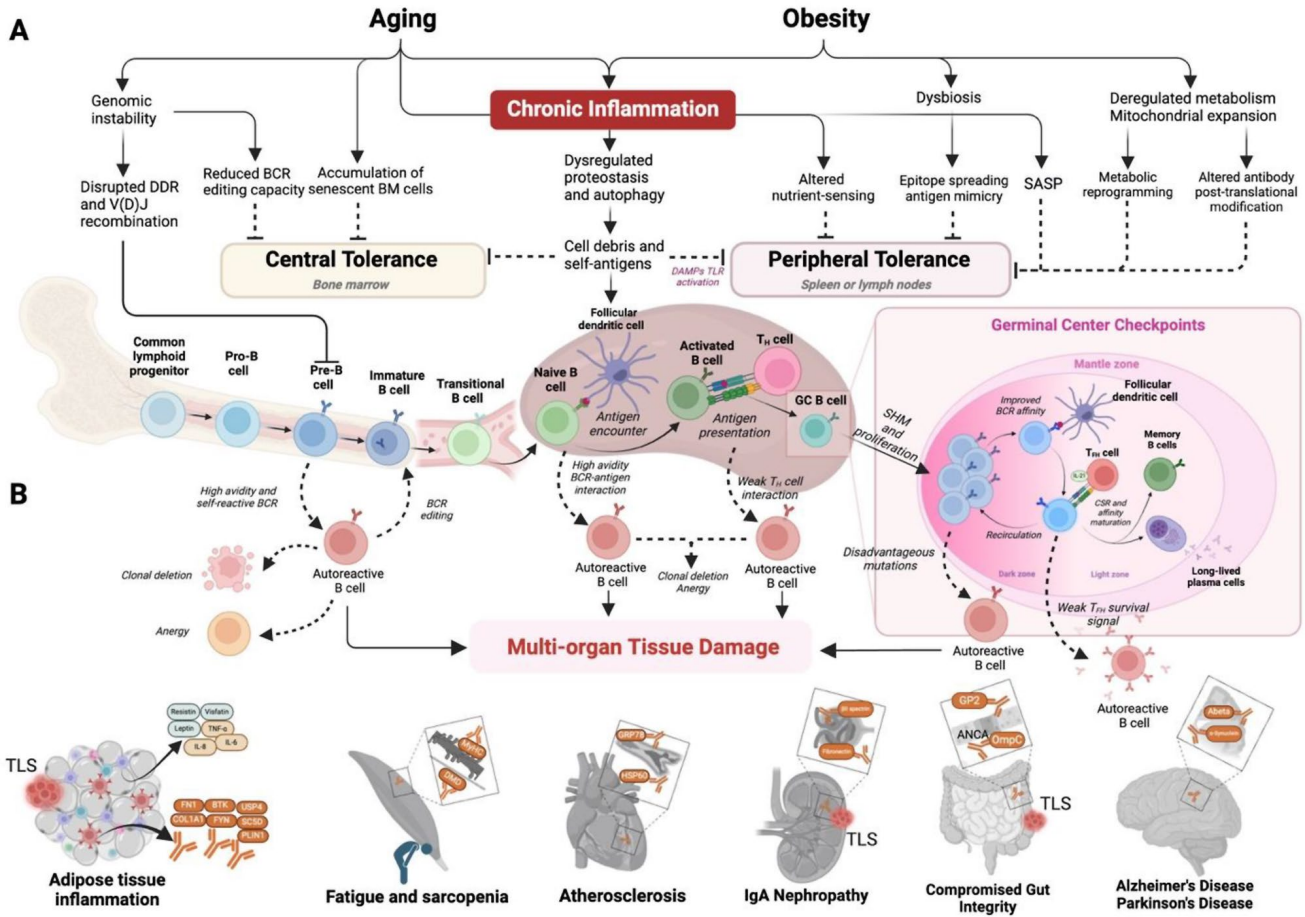
Aging- and obesity-related risk factors contributing to the generation of autoreactive B cells and autoantibodies. **A**) Autoreactive B cells can be generated throughout the B cell development, from hematopoietic stem cells in the BM to various mature, differentiated B cells. In the BM, central tolerance mechanisms, including receptor editing, clonal deletion, and anergy, reduce the autoreactivity of immature B cells, which resulted mostly from the random V(D)J recombination. In the spleen (shown here) and lymph nodes, clonal deletion or anergy remove transitional B cells (spleen) or naïve B cells that strongly bind self-antigens and B cells lacking the costimulatory signals from CD4 + T_H_ cells or CD4 + T_FH_ cells. However, obesity- and aging-related risk factors, including several hallmarks of aging, promote the release of autoantigens and compromise the self-tolerance, facilitating the generation of autoreactive B cells and augmenting the function of existing autoreactive B cells. **B**) In various tissues and organs, including the gastrointestinal tract and kidneys, ectopic lymphoid structures, such as TLS, can potentially provide a niche for autoreactive B cells, facilitating their ability to promote local and systemic inflammation. The resulting pro-inflammatory factors and autoantibodies drive tissue damage in multiple organs, further fueling the ongoing inflammation and systemic diseases. ANCA = anti-neutrophil cytoplasm antibody; Abeta = amyloid beta; ASCA = anti-Saccharomyces cerevisiae antibodies; BCR = B cell receptor; BTK = Bruton tyrosine kinase; COL1A1 = collagen 1 A; CSR = class switch recombination; DMD = Dystrophin; FN1 = fibronectin; FYN = tyrosine-protein kinase Fyn; GC = germinal center; GRP78 = Glucose regulated protein 78; GP2 = Glycoprotein 2; HSP60 = Heat shock protein 60; MyHC = Myosin Heavy Chain; OmpC = outer membrane porin C; PLIN1 = Perilipin 1; SASP = senescence-associated secretory phenotype; SC5D = Sterol-C5-desaturase; SHM = somatic hypermutation; TLS = tertiary lymphoid structures; USP4 = Ubiquitin specific protease 4

The first site where self-tolerance is established is the bone marrow (BM) and is referred to as central tolerance. B cell development begins prior to foreign antigen exposure, whereby immature B cells generate B cell receptors (BCR) through V(D)J recombination, a process involving programmed DNA damage and error-prone repair to increase antibody diversity [26]. Due to the randomness of this process, an estimated 55–75% of early immature B cells can display self-reactive behavior [27]. However, aging-linked genomic instability and chronic inflammation can disrupt the DNA damage response (DDR) and reduce the efficiency of V(D)J recombination [28, 29], leading to higher rates of autoreactive BCRs, though various inhibitory checkpoints act to reduce their frequency [25] (Fig. 1A). A key mechanism of self-tolerance in the central tolerance checkpoint involves negative selection, which relies on the strength of BCR signaling: Pre-B cells with moderate-affinity BCRs are allowed to proceed into immature B cells, whereas Pre-B cells with high-affinity BCRs for self-antigens are directed to undergo receptor editing and clonal deletion, though weak BCR signaling can also allow some Pre-B cells to escape tolerance [30] (Fig. 1A). While central tolerance checkpoints can limit the abundance of autoreactive immature B cells, roughly 40% of newly emigrated B cells into the periphery are self-reactive [27], a rate that can further increase during aging. For example, upon self-antigen binding, murine and human immature autoreactive B cells express 1.5-fold higher C-X-C chemokine receptor type 4 (CXCR4), which is conceived to aid in their retention in the BM for receptor editing [31]. However, elevated plasma C-X-C Motif Chemokine Ligand 12 (CXCL12) during aging [32] can disrupt this retention, potentially allowing autoreactive B cells to enter the periphery.

Transitional B cells exiting the BM migrate to secondary lymphoid organs (SLOs), such as the spleen and the gut-associated lymphoid tissue (GALT), where they undergo positive selection to produce antigen-specific BCRs, while maintaining self-tolerance [33, 34]. Prior to entering germinal centers (GCs), B cells undergo activation at the B-T cell border. This initiates the GC reaction resulting in affinity maturation with help from CD4 + follicular helper T cell (Tfh), follicular antigen-presenting cells, such as follicular dendritic cells (FDCs), and chemokines [35]. As a critical component of peripheral tolerance, self-tolerance in the GCs is achieved through mechanisms, such as clonal deletion, anergy induction, antigen exclusion [36], co-engagement with ITAM expressing Siglecs, activation of ITIM, and other inhibitory receptors and interaction with Tfh cells (Fig. 1A) [33, 37, 38]. Particularly, B cells with higher-affinity BCRs are more effective at presenting antigens to Tfh cells, gaining crucial co-stimulatory cues like CD40L-CD40 and ICOS-ICOSL singular signaling for proliferation [39, 40]. Meanwhile, Tfh-derived IL-21 promotes CSR and SHM via activation-induced cytidine deaminase (AID) and B lymphocyte-induced maturation protein-1 (BLIMP-1) [41, 42], enhancing BCR- and CD40-driven phosphorylation of AKT and S6, key components of the mammalian target of rapamycin complex 1 (mTORC1) pathway [43]. These series of selection, activation, and metabolic reprogramming act as self-tolerance checkpoints, supporting the rapid clonal expansion of GC B cells with proper BCR affinity, while reducing autoreactive B cells to a proportion below 20% [27, 44].

Notably, a pathophysiological form of the GC reaction can also occur in disorganized tertiary lymphoid structures (TLSs) in cases of cancer and autoimmune disease, where they drive opposing outcomes [45] (Fig. 1A). In locations such as the intestinal mucosal surface, TLSs initiate antigen-driven immune responses much like GCs. In the aging bladder and liver, some groups have reported the increased formation of TLSs [46, 47]. Specifically, in the context of kidney injury, aged-associated B cells (ABCs) and a subtype of senescent T cells interact to drive the expansion of TLSs [48]. In autoimmunity, malfunctioning TLS and GC reactions drive the production of pathogenic autoAbs [49], implicating their potential role in driving autoAb production that may exacerbate chronic inflammation during aging and obesity, as discussed below.

## Overview of AutoAb formation during aging and obesity

While the breakdown of autoimmunity checkpoints can lead to autoAb development, additional pathways can also contribute to the loss of self-tolerance. Due to the importance of Tfh cell function on B cell maturation, the loss of, or perturbations in Tfh cells has been implicated in autoAb development [50]. Furthermore, the breakdown of self-tolerance can also arise from T-cell tolerance-related mechanisms, which ultimately affect B cells [51, 52]. During aging, shifts in pro- and anti-inflammatory signals can result in an overall immunosuppressive phenotype from some cells. Aged Tfh cells produce higher levels of IL-10, which is associated with reduced B cell responses to immunization [53]. In addition, dysregulation of CD40 and ICOS signaling in B and T cells in patients with SLE [54] suggests that autoimmunity may be contingent in part on costimulatory signaling. Notably, age-related perturbations in immunometabolism also contribute to an altered T-B crosstalk. For example, splenic B cells from aged mice polarized T cells towards an inflammatory phenotype, and co-culturing young T cells with aged B cells shifted T cell metabolism towards glycolysis, mediated by rises in lactate [55].

Meanwhile, dysfunctional immunometabolism can also directly impact the peripheral tolerance and autoAb development during aging and obesity, where altered insulin receptor expression and function, mTOR signaling, and other nutrient-sensing pathways may influence B cell autoAb production [5, 56]. For instance, Tfh cells induce mTORC1 signaling in GC B cells, leading to positive selection, activation, and migration from the light zone to dark zone. Although transient increases in mTORC1 signaling generated a proliferative boost in B cell proliferation needed for sustained proliferation in the dark zone, constitutive activation of mTORC1 impaired affinity maturation [57]. Hyperactivation of mTORC1 has been observed in aging and obesity [58], giving rise to a potential mechanism whereby constitutive activation of GC B cells by mTORC1 blunts affinity maturation, leading to polyreactive autoAbs. It should be noted that B cell lineage, as discussed above, occurs through many steps throughout multiple tissues. This could imply that altered nutrient signaling induced by aging or obesity in any of these compartments could modulate B cell autoreactivity.

It is becoming clear that autoAb production during aging and obesity is associated with inflammation, which impacts central and peripheral tolerance. For example, aging promotes an increased tumor necrosis factor (TNF-α) production by circulatory B cells in mice and humans [59, 60]. The increased TNF-α levels, primarily found in IgM memory B cells, were negatively correlated with AID mRNA expression, suggesting that increase in some age-related pro-inflammatory cytokines can hinder CSR and SHM [60]. In addition, aberrant macrophage function is also thought to contribute to autoAb development and has been implicated in a variety of autoimmune diseases [61]. The inability to clear damaged cells by macrophages can increase apoptotic antigens that become autoAb targets [62] and impaired macrophage phagocytic or efferocytosis capacity is seen with age [63, 64]. Additionally, macrophages isolated from synovial fluid of patients with rheumatoid arthritis (RA) or from aged individuals express higher levels of pro-inflammatory markers [65], suggesting a correlation between pro-inflammatory states and autoAb production. Thus, dysregulation in the crosstalk between B cells via other immune cells, cytokines, chemokines, and metabolism appear to be potential routes that increase autoAb development. Although more direct evidence is needed to establish causative mechanisms between inflammation and autoAb, modulating inflammation does appear to be involved in the development of autoAbs. Below we discuss in detail the potential mechanisms underlying autoAb production and self-targeting first in obesity and then in aging.

## Mechanisms of AutoAb development in obesity

As a metabolic disease characterized by chronic inflammation, obesity is associated with comorbidities, such as insulin resistance and T2D, and a wide range of immune-mediated diseases, such as multiple sclerosis (MS) and RA [66]. Obesity is also a risk factor in defective immune responses to viruses, such as SARS-CoV-2 [67, 68]. As both the instigator and the product of chronic inflammation, autoAbs have long been known to play a role in mediating the pathogenesis of obesity. Multiple antibodies (e.g. anti-citrullinated protein antibodies (ACPA) and rheumatoid factors (RF) are found positively correlated with obesity [69, 70]. Research has indicated that obesity increased secretion of autoreactive IgG that can target specific self-antigens, which is associated with increased inflammation and dysfunctional glucose metabolism [3, 71]. Additionally, transferring purified serum IgG from high-fat diet (HFD)-fed mice was enough to induce altered glucose metabolism in mice deficient in T-bet + B cells [72]. How obesity can promote the breaching of immune tolerance, the dysfunction of immune checkpoints, and the generation autoAb-secreting B cells has been widely explored, and various mechanisms are discussed below.

### B cell TLRs - drivers of autoimmunity during obesity

Harboring an array of innate and adaptive immune cells, the visceral adipose tissue (VAT) exhibits a shift towards a pro-inflammatory phenotype during obesity [6]. In addition to the pro-inflammatory effect of multiple adipokines, including leptin, resistin, and visfatin [73], adipocyte hypertrophy, hypoxia, and cell death result in the release danger-associated molecular patterns (DAMPs), including “self” protein antigens and cell-free DNA or RNA, which are potent activators of NOD-like receptors (NLRs) and TLRs in macrophages and B cells [66, 74]. For example, obesity induces an increase in VAT T-bet + B cells with marked capacity for IgG2C antibody and the pro-inflammatory chemokine, CXCL10, secretion following glycolipid or TLR7 activation [72]. Meanwhile, HFD-fed mice showed an increased level of autoAb against conserved nuclear antigens, including histone and dsDNA, and obese patients show increased ANA levels, indicating a pathogenic role of TLRs in mediating autoimmune conditions during obesity [74].

The combined TLR and BCR activation by common antigens has been proposed to be a major driving force for the development of autoimmune responses in systemic autoimmune diseases [75]. Numerous common antigens are shared by B cell TLRs and BCRs, including nuclear proteins, citrullinated fibrinogen, cardiolipin, and oxidated phospholipids [75]. In addition, upon BCR binding, self-antigens, such as nucleic acids, can be internalized and translocated to endosomes, where TLR7 and 9 can recognize the same antigens and induce a robust activation of B cells, resulting in a pathogenic GC response, production of autoAbs, and breaching of the peripheral tolerance [76] (Fig. 1A). Indeed, TLR8 KO mice (which can develop TLR7-mediated lupus [77]) fed with a HFD exhibited significantly increased GC B cells, anti-DNA and anti-RNA autoAbs [78]. Consistently, HFD-fed mice lacking TLR7 or TLR9 showed improved glucose tolerance, and TLR7 activation by an agonist can worsen glycemic control [74, 78, 79]. Future work is needed to better define VAT-sourced antigens that can be recognized by TLRs and BCRs.

### Altered nutrient metabolism in B cell dysfunction and AutoAb production in obesity

Obesity is associated with dysregulated lipid metabolism, deregulated nutrient-sensing pathways, and altered mitochondrial energetics, which may contribute to the loss of self-tolerance [80–82]. Through excessive nutrient intake and subsequent meta-inflammation, metabolic reprogramming shifts B cell function towards a pathogenic, autoAb-secreting phenotype [83]. Indeed, B cells from VAT of obese individuals exhibit a hypermetabolic profile and autoAb repertoire, driving the senescence-associated secretory phenotype (SASP). Pathogenic B cells exhibit increased expression of enzymes involved in glycolysis and oxidative phosphorylation (OXPHOS) [84]. In line with this result, splenic B cells from obese mice have increased mitochondrial membrane potential, likely as a response to increased bioenergetic demands [82]. Notably, unlike pro-inflammatory T helper cells, whose bioenergy demand is met by glycolysis, B cell activation and differentiation rely on both glycolysis and OXPHOS [85], the dysregulation of which can lead to autoimmune responses [86]. Furthermore, obesity promotes dyslipidemia and deregulated fatty acid metabolism to facilitate metabolic reprogramming of other immune cells [87]. CD19 + IgD- CD27- B cells (DN) from obese individuals exhibited significantly greater anti-self autoAbs, including adipocyte-specific (anti-SAT) IgG, anti-dsDNA IgG, and anti-MDA IgG, compared to lean controls [16]. Further analysis revealed that the DN B cells had increased expression for enzymes involved in glycolysis, fatty acid oxidation and oxidative phosphorylation, along with higher levels of reactive oxygen species (ROS) [16]. This suggests that during obesity DN B cells have increased metabolic demand, possibly to sustain autoAb production, while supporting their functional requirements. In addition, during obesity B cell activating factor (BAFF) levels have been found elevated in both mice and humans [88–90], where it not only promotes breaking of the peripheral tolerance by suppressing Tregs, but also facilitates the metabolic reprogramming of B cells through increasing both glycolysis and OXPHOS, enhancing antibody production upon LPS stimulation [91], while allowing autoreactive B cells to evade deletion at post-GC checkpoints [92].

### Structural and functional changes in AutoAbs in obesity

Obesity can also cause structural changes to antibodies, predisposing them to become self-reactive. One such modification of antibody function is Fc domain glycosylation, which influences antibody stability, conformation, and effector functions by modulating their affinity for FcγR receptors [93]. For instance, sialic acid and galactose residues are linked to anti-inflammatory activity, while their absence and presence of bisecting N-acetylglucosamine (GlcNAc) residues are associated with pro-inflammatory activity, which is also linked to higher BMI and central adiposity [94, 95]. Structural modifications can also occur to ligands during HFD-induced obesity. Tanigaki and co-workers found hyposialylated IgG2C in mice fed a HFD in addition to IgG in patients with T2D [96]. The hyposialylated IgG2C contributed to inflammation and insulin resistance in part through activation of endothelial FcyRllB [96]. Remarkably, a low-calorie diet significantly reduced the levels of IgG N-glycans with bisecting GlcNAc, which are typically elevated with aging and inflammatory conditions [97]. In addition, Arai and co-workers found that HFD increased the binding of IgM to the apoptosis inhibitor of macrophages (AIM), enabling it to evade Fcα/µR internalization by splenic dendritic follicular cells (FDCs). The IgM-AIM complex results in high levels of IgG in the plasma in obese mice [98]. Additionally, these authors noted higher AIM levels in people with BMIs > 25 with autoimmune disease, suggesting a direct link between obesity and autoimmune diseases [98]. Additional functional changes in antibodies are found in the IgA from HFD-fed mice, which exhibit lower SHM [99], implying an increased polyreactive and autoreactive potential. Therefore, dietary intake may enhance the metabolic adducts and post-translational modifications (PTM) of specific regions on immune cells, leading to structural and functional changes that increase the propensity for autoAb production. These changes may occur through metabolic reprogramming of cellular pathways or modification of the gut microbiota. Future research should focus on identifying the specific PTMs that facilitate the loss of self-tolerance and the mechanisms by which these PTMs arise.

## Mechanisms of AutoAb development in aging

Aging is associated with a decline in immune function that increases the risk for infections, chronic disease, and loss of self-tolerance [100, 101]. The age-related decline in immune cell dysfunction, pro-inflammatory output and decreased antigen specificity is generally referred to as immunosenescence and is associated with endogenous and exogenous stresses that accumulate throughout the lifespan [102]. Key features of immunosenescence include basal chronic inflammation, thymus involution, naive and memory T/B cell imbalances, altered cellular metabolism, and increased senescence associated secretory phenotype SASP [102]. Immunosenescence is a risk factor for ARDs such as Alzheimer’s disease (AD), cardiovascular disease, T2D, and autoimmune diseases [102], and here we propose that it also contributes to dysregulated autoAb production. Aging is characterized by persistent low-grade inflammation, known as inflammaging [103]. Compared with young controls, healthy elderly subjects have higher levels of pro-inflammatory B cells and markers of inflammation [104]. The cumulative exposure to antigens, damage associated molecular patterns, and cellular debris sustains pro-inflammatory pathways [102], which in turn can compromise proteostasis (e.g. by overloading lysosomal pathways, leading to aggregates) and dysregulated mitochondria [105, 106] (Fig. 1A). Aging is also characterized by the dysregulation of nutrient-sensing pathways such as the insulin and insulin-like growth factor (IGF-1) pathways [5, 56]. Phosphoinositide 3-kinase-gamma (PI3Kγ), downstream of insulin and IGF-1, drives ASCs differentiation and potentially T cell-dependent autoAb generation [107]. As a result, insulin receptor signaling may promote autoreactivity, which in turn induces T cell dysfunction [108]. Aside from metabolic dysregulation, IgG antibodies from older humans (> 60 years) have a high propensity to bind to peptides containing two consecutive serine residues at the N-terminus [109], suggesting that autoAbs target selective amino acids. Therefore, the generation of autoAbs with age may be a result of comprehensive stressors placed on the immune system over a lifetime, leading to a set of age-related autoantigens. Driven by the natural progression of aging, immunosenescence is influenced by environmental and lifestyle factors [110]. Studies from mice and humans have shown that aging leads to higher secretion of IgG antibodies with autoreactive behavior [111]. Age-associated intra-tissue accumulation of IgG recently has been found to contribute to senescence development in multiple organs, including spleen, lymph node, hippocampus, lung, and heart [112]. These findings indicate that accretion of cellular defects leading to an immunosenescent phenotype in B cells may ultimately lead to the production of deleterious autoAbs, which may play an underlying role in driving the pathology of ARDs.

### Aging disrupts B cell populations

Aged patients experience impaired B cell proliferation and activation, likely due to defects in their activation threshold. This can lead to a decrease in B cell heterogeneity driven by increased clonal expansion to only select antigens [113]. Moreover, aging induces a shift in the B cell repertoire, towards a B-1 cell phenotype [114]. The increase or expansion in the antigen experienced B-1 phenotype cells may represent a more autoreactive B cell population capable of producing autoAbs. B-1 cells taken from older patients had less spontaneous IgM secretion and no change in IgG secretion, potentially caused by reduced expression of immunoglobulin secreting transcription factors [115]. At the cellular level, aging is associated with decreased expression of the transcription factor, E47, necessary for B cell differentiation, and AID, which is required for SHM and CSR [116, 117]. The decrease in E47 and AID was associated with remarkably lower levels of memory B cells, and higher percentage of naive B cells [117]. As such, aging populations have blunted neutralizing responses to vaccination [118] and present disease modifying autoAbs, which exacerbate pathogen-induced inflammation [119]. Additionally, aged B cells have a reduced ability to differentiate self from non-self-antigens because of the oligoclonal expansion of B lymphocyte subpopulations rich in antigen-experienced cells. These subpopulations of B cells in mice have age related B cell features (CD21low/-, CD23low/-), and others express CD5, enabling the production of low-affinity antibodies [120]. CD5 + B cells are associated with various autoimmune diseases and can produce natural antibodies that typically target infectious agents. With age these natural antibodies may display a repertoire that is indicative of autoreactivity [121]. This characteristic is significant in the context of autoAb generation, as aging induced modifications to B cell populations can initiate an autoimmune response [122].

Related to these findings, the loss of immunological tolerance to auto-antigens in aging is also associated with an increased frequency of aged associated B cells (ABCs in mice) and double negative (DN2 in humans) populations [123, 124]. ABCs are considered antigen-experienced B cells, identified as CD21- and CD23- B cells, which also contain fractions of T-bet+, CD11b+, and CD11c + cells [108, 125, 126]. Heterogeneity in surface marker expression has been observed in a mouse model of SLE, suggesting ABC-like subsets can arise in autoimmune diseases [127]. ABCs are enriched in patients with autoimmune disease and appear in in-vivo models of autoimmunity, where they have been observed to drive inflammation, producing autoAb [126]. ABCs are thought to arise from the exhaustive expansion of the follicular B cell pool that occurs naturally through aging [125], through signaling pathways that induce T-bet expression [128] and are responsive to TLR7/9 stimulation [125]. The transcription factor ZEB2 was recently found to be required for ABC formation and production of IgG2c, an inflammatory isotype of ABCs [129]. This unique population of B cells is characterized as being pro-inflammatory and autoreactive [130] while producing a unique secretome and transcriptome in patients with RA [131]. In humans with SLE, the abundance of ABCs correlates with disease severity [132], suggesting a potential pathogenic function of this B cell subset. Notably, ABCs from old mice secrete higher levels of IgG antibodies against MDA and adipose tissue-derived antigens [111], suggesting that during aging, ABCs contribute to chronic inflammation and autoAb production. ABCs have been shown to accumulate in the spleen, liver, and salivary glands in old mice, where they induce inflammation and suppress B cell lymphpoiesis [133, 134]. These results suggest that ABCs can exert detrimental functions throughout a variety of tissues, while impairing B cell lineage progression. Future research will need to determine the full spectrum of ABCs induced pathology in immune and non-immune tissue.

### Temporal dynamics of AutoAb during the lifespan

AutoAb development observed during aging appear to follow temporal patterns becoming enriched during ARDs. For example, one study found that the abundance of autoAbs was significantly greater in people aged 65 years and older compared to those younger than 45 [135]. AutoAb abundance was also greater in those diagnosed with AD, MS, and Parkinson’s disease (PD) [135]. Yin and co-workers found 3 waves of autoimmune signatures corresponding to ages 30, 50, and 62. 162 autoAbs were shared between the 30- and 62-year-old age groups, while each age group had 546 and 118 unique autoAbs, respectively [136]. Interestingly, those in the 50-year-old group had 12 unique autoAbs, suggesting that throughout aging, autoAb development occurs in waves, rather than linearly. Longitudinal sampling from 35 patients within the Swiss HIV Cohort Study revealed the presence of autoAbs for INFɑ, β, and ⍵ during the 6th decade of life, on average [137]. Interestingly, autoAb to IFN-I may have occurred in subjects with reduced IFN-stimulated gene (ISG) levels, indicating innate immune dysfunction, or people with pre-existing autoimmunity who reacted against IFN-I injection therapy; these data suggest that aging, along with other factors, including pre-existing or concomitant loss of self-tolerance plus past exogenous IFN-I therapy (i.e., past exposure to antigen) may contribute to IFN-I autoAb development [137]. A recent multi-omics analysis of 108 individuals found two crests of dysregulated molecules that occurred at approximately the age of 40 and 60 [138]. The biological function modules associated with crest 1 (aged 44) included skin/muscle, cardiovascular disease, alcohol, lipid and caffeine metabolism, while those associated with crest 2 (age 60) were skin/muscle, cardiovascular disease, kidney function, immunity, carbohydrate, and caffeine metabolism [138]. Notably, functional analysis indicated that pathways related to antioxidant activity, oxidative stress, and oxygen carrier activity showed marked nonlinear changes in the age of 60s, while pathways related to mRNA destabilization, mRNA processing, and macroautophagy showed marked nonlinear changes in the age of 70s [138]. These changes may provide insights into the formation of age-related pro-inflammatory environments that trigger generation of autoantigens and potentially autoimmune responses in specific decades of life. A similar study investigating the proteome also found crests of aging proteins at 34, 60 and 78 years. These crests were largely defined by unique protein signatures relating to cardiovascular disease, Alzheimer’s disease, and Down Syndrome enriched in middle age (60) and old (78) age [139]. Additionally, two-thirds of the proteins that changed with age also changed with sex [139]. While molecular events triggering these waves of aging and the associated autoAb development have not been determined in a temporal order, positive correlations between sex hormones, renal, and immune aging were found in a cohort of individuals aged 20–45 [140]. Other patterns of nonlinear aging have been observed in DNA methylation dynamics of mice colons [141]. Therefore, the nonlinear waves of aging may be a result of multiple contributing factors, including hormones, shifts in metabolism, and epigenetics, the alteration of which can eventually contribute to a niche of chronic inflammation, favoring the pathological actions of autoAbs. The autoAb signatures corresponding to specific decades of life could hence be used as predictive markers for aging to determine the onset of ARDs. These data also suggest specific decades of life when interventions may be more crucial compared to others. Future research needs to determine at what point throughout the lifespan interventions provide the most robust protection.

## Effector actions of autoantibodies in obesity and aging

Once generated, autoAbs exert their pathological functions through a variety of mechanisms that lead to prolonged immune activation, inflammation, and tissue damage. Some of these mechanisms include binding to Fc receptors (FcR), activating complement, neutralization of antigens, opsonization, and cell-specific depletion and clearance, which all modulate inflammation [13, 142, 143]. Features of premature immunosenescence, such as accelerated telomeric attrition and excessive production of pro-inflammatory cytokines, have been observed in some autoimmune diseases, such as RA [144]. Notably, increased titers of autoAbs are detected before the onset of the disease [145], indicating a potential early role of autoAbs in promoting immunosenescence and chronic inflammation. For example, the immune complexes ACPA-IgG activate macrophages via TLR4 and FcR, while complement activation by autoAbs can boost inflammation in the synovium [146]. These events create an inflammatory niche through the production of age-related cytokines, such as TNF and IL-6 [147, 148]. Similarly, IgG accumulated during aging can prime macrophages to a senescent state through IgG FcR, as indicated by increases in SA-β-Gal activity, p21 and inducible nitric oxide synthase (iNOS) expression and the production of nitric oxide, all of which are considered hallmarks of inflammatory macrophages [112]. In another example, autoAbs increase in patients with long COVID [149], along with prolonged complement activation leading to persistent immune activation and T-cell exhaustion, risk factors of immunosenescence [150–152]. We next discuss how such effector mechanisms of autoAb function are potentially influenced with a focus on obesity and/or aging.

### Effector actions through dysregulations in the complement system

As one important axis of the antibody effector function, the regulation of antibody-mediated complement activation becomes impaired during inflammaging, potentially due to alterations in complement protein levels and the impact of cellular senescence [153, 154]. Studies have shown that levels of key complement components, such as C3 and C4 are elevated with age [155], while higher C5a levels are correlated with disease severity in AD patients [156], implicating a role of complement factors in contributing to an enhanced chronic inflammatory state. BM-derived B cells from lupus-prone mice expressing the human complement receptor 2 (hCR2), demonstrated increased susceptibility to apoptosis and decreased ANA [157]. The presence of C3 was associated with a down-regulation hCR2, suggesting that C3 may interfere with central tolerance mechanisms, enhancing the probability of positively selecting autoreactive B cells [157]. Beyond its ability to drive aging via broad crosstalk with hallmarks of aging, such as altered metabolism and mitochondrial functions [158], often overlooked is the potential role of complement activation in modulating the autoreactivity of GC B cells. Specifically, it has been shown that complement-opsonized immune complexes are delivered by follicular B cells shuttling from the marginal zone to the FDCs in GCs [159], where antigen-specific B cells then capture the FDC-processed antigens and present them to CD4 + T cells, acquiring survival signals [160]. During aging, a decline in the phagocytic efficiency of immune cells, such as macrophages, can result in the accumulation of complement-opsonized debris and apoptotic cells, potentially containing self-antigens [161]. The binding of these complement-coated immune complexes to self-antigen-specific B cells can eventually lower the activation threshold for these cells, allowing them to bypass normal tolerance mechanisms [162].

### Effectors actions of antibody-dependent enhancement through FcR signaling

In parallel, the FcRs are also critical determinants in autoAbs effector function by modulating immune responses, such as inflammation and immune cell activation, which can either enhance or suppress autoimmunity. Increased inflammatory signaling by effector cell activation via FcRs is seen in the pathogenic immune response, termed antibody-dependent enhancement (ADE). FcR engagement with non-neutralizing or sub-neutralizing antibodies promote viral infection, intensifying diseases [163, 164]. A relevant component of ADE lies in the context of pre-existing immunity typically induced by prior vaccination or infection, leading to enhanced FcR signaling, driving the expression of proinflammatory cytokines [165]. It is therefore not surprising that FcR are being studied as a potential mechanism mediating autoimmune disease, where autoAbs can interact with a variety of FcRs leading to ADE [166, 167]. IgG autoAbs taken from patients with SLE were shown to opsonize late apoptotic Jurket cells, inhibiting their ability to be phagocytized in a FcγR-dependent mechanism [168]. While the consequences of increased apoptotic cells were not investigated, one could speculate this could be potentially deleterious and disease-escalating. Fukue and co-workers found that the disease-modifying anti-rheumatic drug, abatacept, downregulated FcγR1 expression on monocytes in patients with RA. The decrease in FcγR1 expression led to suppression of pro-inflammatory cytokine production in ACPA immune complex treated cells [169]. Excessive engagement of FcRs through autoAb-dependent mechanisms appear to promote chronic inflammation, which could be involved in aberrant immune responses seen during obesity and aging. FcγR -/- mice fed a HFD had less weight gain, decreased insulin resistance and lower expression of pro-inflammatory genes compared to wild type controls [170]. In aging, increased IgG hinged on FcRn mediated recycling by macrophages [171]. Ablation of FcRn lowered adipose tissue IgG, improved survival and glucose metabolism, and facilitated a beiging phenotype in eWAT (epididymal white adipose tissue) [171]. FcR signaling was shown to activate a proliferative response in microglial cells when mice were administered anti-myelin oligodendrocyte glycoprotein (MOG) antibodies [172]. While pathogenic or therapeutic outcomes were not studied, the induction of microgliosis was amplified in mice harboring a constitutively active, plasma membrane-bound Brutons tyrosine kinase (BTK) [172]. This is interesting because reports from our laboratory and others have shown BTK as one of the top autoantigens in serum [3] and stromal vascular fraction [71] in obese individuals. This may mean that a portion of autoAbs seen in obesity could be involved in a compensatory mechanism to mitigate the diseased state. These studies suggest that FcR signaling is an important mediator in autoreactivity. Understanding how immune complexes interact with FcR in obesity and aging will be of high importance for future research.

### Natural killer cells augment inflammation-driven AutoAb effector actions

Interestingly, as an effector of antibody-dependent cellular cytotoxicity (ADCC), NK cells in aged murine BM show a 2-fold expansion, contributing to reduced B cell lymphopoiesis via TNF-α-mediated inhibition of E47, a transcriptional regulator of B lineage commitment [173]. NK cells also show subset-specific effects in mediating tissue inflammation and senescence. For instance, IgG levels in SS patients and seropositive patients with early RA (i.e. positive for ACPA and/or RA) are correlated with the ratio of CD56^bright^ to CD56^dim^ NK cells, where the reduction in the circulating CD56^dim^ NK cells contributes to accumulation of these cells into glands (and joints in the case of RA), enhancing focal immune injury and eventually systemic inflammation through secretion of pro-inflammatory cytokines (e.g. IFN-γ and TNF-α) [174–176]. While chronic inflammation driven by autoAbs plays a contributing role in the induction of immunosenescence, more work is needed to determine the relative contributions of obesity- or aging-associated autoAbs linked to NK cell-mediated ADCC.

## Autoantigen targeting in obesity and aging

### Immunogenicity of autoantigens in obese humans and mice

The immune responses mediated by autoAbs rely on the immunogenicity of autoantigens, which can be generated through obesity-associated events such as PTMs [177], dysregulated lipid metabolism [178], cellular stress, and perturbed proteostasis [179] (Fig. 1A). One of the top IgG antibody targets in obese human males with impaired insulin sensitivity, and even in studies of type 1 and 2 diabetes, is glial fibrillary acidic protein (GFAP), an intermediate filament protein expressed primarily in astrocytes and non-myelinating Schwann cells [3, 180–182]. Increased astrogliosis and GFAP immunoreactivity increased in the hypothalamus in mice fed a HFD, which formed distinct patterns around microvessels [183]. The close proximity to the vasculature may indicate a release of GFAP by activated astrocytes during obesity-associated central nervous system (CNS) inflammation. Previous reports confirmed that GFAP can be recognized by autoreactive IgG antibodies that can induce abnormal glucose metabolism [3, 184]. Given its correlation with the onset of T2D, GFAP has been used as a T2D biomarker in both humans and mice [180]. However, due to its location in immune privileged sites like the brain, targets like GFAP likely require an inflammatory milieu to break tolerance. In fact, many autoantigens that have been discovered linked to insulin resistance, which precedes T2D, are intracellular antigens present across many tissues (Table 1), and would likely need the correct inflammatory milieu to unmask their antigenicity to break tolerance [3]. How such inflammatory milieu is formed and how additional autoAb-autoantigen interactions contribute to age-related pathologies in the CNS and other tissues will be detailed in the later section.

**Table 1:** Autoantibody Isotype and autoantigens found in obesity and aging

Antibody Isotype	Autoantigen(s)	Condition	Reference
IgG	-Linked to insulin resistance: GOSR1, BTK, GFAP, ASPA, NIF3L1, PGD, ALDH16A1, KCNAB1, RNA polymerase, GSTA3 -Linked to insulin sensitivity: CTNNA1, CDC37, LGALS14, BM88, NCBP2, PDDC1, ALS2CR8, PAFAH G SUBUNIT, XRCC4	Obesity (DIO mice)	[3]
IgG3	PDIA3	Obesity (HFHF diet mice)	[15]
IgG	MDA and adipocyte derived protein antigen	COVID-19/Obesity (patients)	[67]
IgG	USP4, FYN, C5SD, FN1 COL1A1, BTK, GSTA3 FAD, ApoB, PIK3CB ECHDC3, KCNA10, ASPA H2AC, CARM1 hnRNPA2B1	Obesity (human subcutaneous adipose tissue)	[71]
IgG, IgA, IgM, IgE	Type I Interferon (IFN I)	COVID-19/aging (patients)	[119, 315]
IgG	RSPH10B2, CARNMT1, PEX16, CPB1, GLUL, RSP6KA6, RPS6KA3, MRPL52	Aging (patients)	[136]
IgG	pSMAD2 and pSMAD3	Aging (mice)	[171]
IgG1	GFAP and iba-1 reactive	Obesity (HFD mice)	[184]
IgG	Anti-dsDNA and ANA	Obesity (lupus-prone mice)	[189]
IgG	EPHX2, GDH1, HMGCS2, CAT, ALDHE2	Aging (keratin knock-out mice)	[207]
IgG1, IgG4	cTnT, TnT3	Aging (cognitively impaired patients)	[218]
IgG	ATCAY. HIST1H3F, NME7, NOL3, PAIP2	Aging (cognitively impaired patients)	[279]
IgG	Glycosylated human serum albumin	Aging (smokers)	[316]
IgG, IgM, IgA	CRLS1, dsDNA, ssDNA, RF	Aging (patients)	[317]
No isotype stated	GAD65	T2D (patients)	[318]
IgG	RhoA and CASP3	Maculopathy-T2D (middle-aged patients)	[319]
IgG	Glutamate	Aging (cognitive impairment)	[320]
IgG	Amyloid beta (Aβ); Aβ1–42	Aging (patients)	[321]
IgG	Anti-cell extract, ANA,	Aging (non-human primates)	[322]

In addition, products related to altered metabolism can also contribute to the immunogenicity of autoantigens during obesity. For example, elevated levels of advanced glycated end product (AGE)-modified proteins, such as serum albumin, are observed in circulating immune complexes of hyperglycemic plasma from patients with diabetes [185]. This observation is consistent with the findings that increased levels of circulating antibodies against AGE-modified proteins are found in patients with coronary atherosclerosis [186]. Notably, RA patients with T2D also exhibit high serum levels of autoAbs against AGE-modified IgG, exacerbating the disease severity [187]. Furthermore, obesity-associated dyslipidemia enriches the repertoire of self-lipid antigens, including various glycosphingolipids (GSLs), recognized by CD1d invariant natural killer T cells (iNKT) [187]. These iNKT cells are capable of promoting B cell production of autoAbs via cognate or non-cognate manners [71], as seen in a murine model of human lupus with heightened levels of anti-lipid IgG antibodies [188].

In addition to products of metabolic dysregulation, self-proteins from obesity-associated chronic inflammation are also candidates for autoantigens, as they are released from injured tissues during chronic inflammation. Antigens resulting from obesity-associated inflammation, including MDA and adipose-derived antigens also induce IgG autoAb production, interfering with the normal production of neutralizing antibodies upon viral challenges in obese SARS-CoV-2 patients [67]. Antigenic targets from chronically inflamed VAT will be discussed in more detail below. Notably, antigens from preexisting autoimmune conditions show an amplified antigenicity during obesity. For example, HFD feeding in MRL-lpr (lymphoproliferation) mice is accompanied by increased plasma levels of anti-dsDNA and ANA IgG antibodies, leading to an exacerbated SLE phenotype [189].

Taken together, these findings show that obesity may not only induce metabolic reprogramming in B cells and other immune cells but also facilitate structural and functional changes in autoAbs and autoantigens, which may also increase during low-level tissue damage with chronic inflammation. These events can worsen metabolic outcomes and favor the induction of autoimmune responses. In return, these changes can accelerate immunosenescence, weakening the immune system, and increasing susceptibility to infections and autoimmune diseases.

### Immunogenicity of autoantigens in aging humans and mice

The aging process is driven by interconnected cellular, molecular, and systemic events, termed the hallmarks of aging [190], that ultimately lead to the breakdown of homeostasis and mortality [191]. In 2023, the hallmarks of aging were updated to include 12 interdependent features of cellular alterations that contribute to the decline in organismal function [190]. Our laboratory recently explored the hallmarks of aging through the view of immunity, by adding the contribution of chronic inflammation to each hallmark, integrating the immune system with all the features of aging [5]. Autoantigens found in aging populations are associated with pathways involved in the hallmarks of aging. Here we further explore the immune system’s participation in the hallmarks of aging with a particular emphasis on autoAbs and autoantigens.

During aging and ARDs, the ribosomal protein S6 kinase (RPS6KA6) has been reported as a top autoantigen associated with serum IgG autoAbs [136]. Additionally, eukaryotic translation initiation elongation factor 3 (eIF3) and ribosomal P proteins were autoantigen targets of autoAbs in autoimmune cerebellar ataxia, an age-associated autoimmune disease, and in patients with SLE [192, 193]. Such reductions in translational capacity and ribosome biogenesis have been shown to occur during aging, potentially leading to protein aggregates and perturbed protein translation [194, 195]. Dysregulated proteostasis also arises when there is dysregulated autophagy. While there is limited evidence that autoAb target autophagy-related proteins during aging, there are several links between autophagy and autoimmune diseases [196]. Individuals with aged-related macular degeneration (AMD) have several circulatory autoantigens related to autophagy that are targets of autoAbs [197]. In another study, among patients with wet or dry AMD, the top autoantigens found in their blood included alpha-synuclein, annexin V, heat shock protein 10 (HSP10), and GAPDH [198]. The range of antigens found in this study suggests that autoAbs are targeting proteins involved in protein folding, metabolism, and apoptosis. Mice lacking the immunity-related GTPase, IRGM1 gene, manifested an aberrant immune response characterized by type 1 interferonopathy, similar to the autoimmune disease Sjorgen’s syndrome [199]. The accumulation of type 1 IFN was due to impaired mitophagy, another feature of aging, which increased cytosolic mtDNA and mtRNA initiating an autoimmune response [199]. Although additional research is required to identify the autoantigen targets associated with aging, these studies underscore the interaction of autoAbs and their role in autophagy and age-related processes.

Increased fibrosis is associated with aging and is manifested in many ARDs, such as chronic kidney disease, fatty liver disease, idiopathic pulmonary fibrosis (IPF) and heart failure [200]. Analysis of lung tissues taken from older patients (65 ± 10 years of age) with IPF showed increased senescent biomarkers compared to controls. Additionally, treating fibroblasts with culture media taken from senescent cells resulted in greater expression in pro-fibrotic genes [201], suggesting that senescence factors can promote fibrosis. IgG antibodies in adipose tissue increase linearly with age in both mice and humans and may instigate a pro-fibrotic environment [171]. Treating young mice with IgG from old mice to mimic aging increased the expression of genes related to tissue fibrosis [171]. These authors then determined that IgG treatment led to higher levels of phosphorylated MEK, ERK1/2, and RAS GTP, which upregulates TGF-β in BM-derived monocytes and promotes fibrosis [171]. Mice deficient in B cells were protected from IgG-induced adipose tissue fibrosis, suggesting B cells can communicate with other immune cells to drive tissue remodeling [171]. In aging patients with different subtypes of pulmonary fibrosis, shorter leukocyte telomere lengths were observed compared to healthy age-matched controls [202]. Idiopathic pulmonary fibrosis was found to have one of the highest rates of telomerase mutations [203]. These studies provide evidence that pro-fibrotic environments are associated with telomere attrition.

In an autoimmune fibrotic disease, such as systemic sclerosis, characterized by accelerated aging, patients exhibit several hallmarks of aging such as mitochondrial stress, chromosome instability, and shortened telomere length [204]. Furthermore, a subset of patients diagnosed with systemic sclerosis had higher autoAbs targeting the telomere/shelterin complex, including the telomeric repeat-binding factor 1 (TERF1) as the most common autoantigen [205]. Another study discovered 8 additional autoantigens, including TERF2, associated with telomeres or telomerase in systemic sclerosis patients [206]. Analysis of peripheral blood leukocytes in systemic sclerosis patients revealed a negative association between TERF1 autoAbs and telomere length. These authors screened an additional cohort of patients with idiopathic pulmonary fibrosis and discovered that 11 out of 152 patients had TERF1 autoAbs [205], suggesting a potential relationship between telomere dysfunction, fibrosis, and the immune system. Notably, TERF1 levels decrease during aging in mice and humans, and a study using gene therapy to transiently overexpress TERF1 in mice significantly prevented age-related decline in neuromuscular function, glucose tolerance, and cognitive function [207, 208]. While studies that characterize TERF1 autoAb levels in healthy aging individuals are lacking, the correlation between TERF1 autoAbs, telomere shortening, and potentially age-adjusted telomere decline highlights their possible role in aging and immunosenescence. This area may benefit from further research, as understanding these connections could enhance our knowledge of telomere biology in aging and ARDs. Finally, aged mice lacking the gene for keratin 8 displayed high titers of autoAbs against the mitochondrial proteins, including glutamate dehydrogenase, catalase, mitochondrial HMG-CoA synthase, and aldehyde dehydrogenase E2 [209]. Therefore, mitochondrial health appears to rely on the integrity of intermediate filaments like keratin, the loss of which may promote oxidative stress and mitochondrial dysfunction. These studies suggest that alterations to the cytoskeletal network are associated with elevated autoAbs in pathways that are connected to some of the hallmarks of aging. More work will be needed to determine if changes in environmental forces associated with fibrosis and aging also facilitate autoantibody mediated production [210].

## Autoantigen targets during obesity and aging

### Skeletal muscle

Skeletal muscle accounts for roughly 40% of total body weight and plays a vital role in movement, posture and metabolism [211]. Aging and obesity compromise the regeneration of skeletal muscle and are accompanied by the loss of muscle mass and function [212, 213]. Diseases such as diabetes impact multiple cell populations within the skeletal muscle niche, resulting in a decline in muscle regeneration [214]. Single-cell RNA sequencing of human skeletal muscle has shown that aging is characterized by increased B and T cell populations, while M2-like macrophages decline [215]. In another human study, using single nuclear RNA sequencing study, antigen presentation, IFNγ responses, and complement cascades were upregulated in immune cells or fibro-adipogenic progenitors in old muscle, raising the possibility of classical antibody-dependent complement fixation signatures [216]. Additionally, blunted muscle hypertrophy and higher expression of senescence markers were found in old mice after surgery-induced muscle hypertrophy [217]. These results suggest that aging muscle is met with remodeling of the immune system and age-induced senescence which could be a contributing factor to sarcopenia (Fig. 1B).

Specific autoantigens have been investigated in skeletal muscle in the context of autoimmune disease, like Myasthenia Gravis (MG) or myositis, but less so in the context of obesity or aging. In MG, almost 90% of patients have IgG1 acetylcholine receptor autoantibodies and about 10% of patients have IgG4 muscle-specific kinase autoantibodies [218]. Myosin heavy chain has also been shown to be an autoantigen in skeletal muscle, and mice immunized with purified skeletal muscle myosin developed experimental autoimmune myositis [219, 220]. Additional autoantigens found in myositis include Histidyl tRNA synthetase (HRS/Jo-1), Mi-2, U1-70kD, and the catalytic subunit of DNA-dependent protein kinase (DNA-PKcs) [221]. Interestingly, these autoantigens are also expressed during skeletal muscle regeneration, suggesting that different stages of skeletal muscle injury/recovery may pose greater risks for autoAb pathogenicity. Furthermore, regeneration is impaired in aged skeletal muscle, which may be in part a result of dysregulated autoAb accumulation. For example, IgG-mediated autoimmunity has been proposed to be involved in the pathogenesis of sarcopenia, including potential targeting of cardiac troponin T in skeletal muscle [222]. Specifically, an increased abundance of IgG1 and IgG4 in skeletal muscle was associated with declined physical performance in humans and mice [218]. IgG co-localized with markers of the complement pathway, necroptosis, and loss of dystrophin [218] further linking autoAbs and skeletal muscle pathology. Going forward, it will be interesting to investigate whether subsets of obesity, including patients with sarcopenic obesity, which shows overlap with muscular features of aging, has any overlap in autoantibody repertoires.

### Cardiovascular tissue

The prevalence of cardiovascular disease (CVD) increases during aging [223], in association with other risk factors such as smoking, obesity, hypertension, diabetes, sedentary lifestyle, poor dietary choices, and socio-economic status [224]. A major cause of CVD is atherosclerosis, which is now considered an immune-mediated etiology [225]. Foamy macrophages found in early and advanced atherosclerotic lesions express senescence markers [226], suggesting a role for immunosenescence in the pathology of atherosclerosis. Additionally, several autoantigens involved in the development of atherosclerosis have been detected (Fig. 1B). Glucose-regulated protein 78 (GRP78), an endoplasmic reticulum (ER) chaperone, can localize to the cell surface under periods of ER stress. In the early stages of atherosclerosis development, cell surface localization of GRP78 and increased anti-GRP78 autoAbs are seen, leading to NF-κB signaling in endothelial cells, and accelerating atherosclerotic lesion growth [227]. Another autoantigen found in atherosclerosis is heat shock protein 60 (HSP60). Injecting apolipoprotein E (ApoE)-deficient mice with anti-HSP60 autoAbs led to increased lesions in the aortic sinus [228]. Lorenzo and co-workers determined autoAbs that were reactive against atherosclerotic plaques. Interestingly the reactivity patterns were diverse, indicating that the plaques contained multiple antigenic targets [229]. One particular autoantigen studied was aldehyde dehydrogenase 4 family member A1 (ALDH4A1). These authors showed that anti-ALDH4A1 autoAbs delayed atherosclerosis plaque formation when infused into low-density lipoprotein receptor-deficient (Ldlr-/-) mice [229]. Other commonly studied targets during atherosclerosis include potentially pathogenic IgG antibodies against oxidation specific epitopes, and atheroprotective natural IgM anti-phosphorylcholine types [230]. Finally, an emerging antigenic target of adaptive immunity during atherosclerosis includes ApoB, though current research has been skewed more towards T cell targeting of ApoB than B cell targeting [231]. In summary, during CVD, several autoantigens can exacerbate disease, while others become targets of autoAbs, potentially delaying disease.

### Adipose tissue and liver

The adipose tissue has a remarkable capacity to store excess energy, while also functioning as a regulator of hormones, appetite, immune responses, and thermogenesis [232, 233]. During aging, brown (BAT) and subcutaneous adipose tissues (SAT) decrease with a concomitant accumulation in VAT and ectopic lipids [234]. In addition to adipocyte remodeling, immune infiltration into the VAT increases resulting in a pro-inflammatory state during aging and obesity [6]. Obesity suppresses the secretion of regulatory adipokines, such as adiponectin, further leading to whole-body metabolic impairments [235]. Increased senescent preadipocytes were observed in VAT isolated from aged donors [236]. The senescent preadipocytes were characterized as being pro-inflammatory and capable of inducing inflammation in neighboring cells [236]. Therefore, aging and obesity promote a detrimental remodeling of adipose tissue, which may enhance immunosenescence. Adipose tissue has been found to not only be a source of inflammation but also the production and secretion of autoAbs [71, 237] (Fig. 1B). Indeed, self-antigens may be released from VAT through additional mechanisms linked to aging and obesity, including hypoxia, cell cytotoxicity, metabolism, hormones and DNA damage [71] (Table 1). Patients with general acquired lipodystrophy had anti-Perilipin-1 IgG antibodies that were capable of inducing lipolysis in-vitro [238]. Treating VAT cultures from obese patients with IgG-ACPAs, commonly seen in RA, led to increased expression of pro-inflammatory markers, while suppressing the expression of genes involved in the insulin signaling pathway [239].

In addition, obesity is associated with metabolic liver disease, driven in part by lipotoxicity, immune-mediated inflammation, and glucotoxicity, inducing liver damage [240–242]. Recently, a high-fat high-fructose (HFHF) diet was shown to promote MHC-II presentation of epitopes from protein disulfide isomerase family A member 3 (PDIA3) [15]. Anti-PDIA3 AutoAbs showed isotype switching from IgM to IgG3, and transfer of PDIA3-specific antibodies could exacerbate hepatocyte death. Increased humoral responses to PDIA3 were also observed in patients across a diverse array of chronic inflammatory liver conditions, such as autoimmune hepatitis, primary biliary cholangitis, and T2D, further supporting PDIA3 as an important liver-derived autoantigen during liver inflammation. Intriguingly, a novel subset of adipose tissue macrophages, termed inflammatory and metabolically activated macrophages (iMAMs), was found in obese humans and mice and enriched in PDIA3 expression [243]. PDIA3, in turn, exerts redox control on RhoA activity, strengthening the pro-inflammatory and migratory capacity of iMAMs through RhoA-YAP signaling, contributing to worsened VAT inflammation and metabolic outcomes [243]. This finding highlights an underappreciated role of the mechanosensing immune cells [244] and PDIA3 self-antigens in mediating a mechano-redox control of inflammation, especially during obesity and aging, where the VAT and the liver exhibit increased tissue stiffness due to hypertrophy, extracellular matrix accumulation, and fibrosis [245–248].

### Spleen

As a critical extramedullary hematopoietic immune organ, the spleen maintains the homeostasis of the immune system in cooperation with the BM. However, aging is associated with an altered immune landscape in the spleen as it houses autoAb-producing B cells and other autoreactive immune cells that favor a pro-inflammatory and pro-immunosenescent phenotype [249]. The ABCs, as introduced above, are autoAb-secreting B cells often found to accumulate in aged or autoimmune-prone mice [125, 128]. For example, ABCs expand in spleens of MRL/lpr mice and produce autoAbs, such as anti-nucleosome and anti-RNA IgG, upon stimulation of TLR7 and TLR9 [127]. Other autoAbs specific to self-antigens include RF and ds-DNA are linked to conditions like SS, SLE, and malaria anemia [126]. The expansion of ABCs and autoreactive B cells in general might be related to impaired regulatory B cell (Bregs) function in the spleen. Specifically, Bregs can secrete IL-10, IL-35, and TGF-β to suppress CD4 + T cell function (including the pathogenic Th17 cells in the spleen that can promote autoAb production [250] and Tfh cell differentiation [251, 252] in patients and mice with autoimmune diseases, potentially preventing autoAb formation [253]. However, whether Bregs can directly suppress ABC-induced inflammation during aging and obesity needs further investigation.

Obesity fosters a chronic inflammatory state that promotes the survival and activation of splenic B cells. In DIO mice, the spleen often contains a lower percentage of IgM + IgD − cells and shows reduced spontaneous IgM production but increased IgG secretion, indicating that HFD-induced obesity induces a systemic humoral immune response [3]. T-bet + CD11c + B cells are also found to increase in spleens of HFD-fed mice and humans with higher body mass index [72]. There is a marked correlation between the proportion of T-bet + CD11c + B cells in the spleen and serum levels of IgM and IgG2c in both WT and Cd1d1-/- mice, suggesting that the expansion of these B cells and increased antibody production in obesity are supported by iNKT cells, an inflammatory subset capable of producing IFNγ [72]. Taken together, these findings show that the spleen plays an important role in autoAb-driven inflammation, as seen in autoimmune diseases, aging, and obesity. Mechanistically, the extrafollicular B cell activation and reduced GC responses lead to heightened production of pro-inflammatory cytokines and autoAbs, which are risk factors for immunosenescence [254–256].

### Gastrointestinal

An emerging area of research is the interaction between the microbiota and host antibody production. Specific gut microbes can be a source of antigenic mimicry, induce autoreactivity in immune cells, or secrete metabolites that control inflammation [257]. Aging and obesity are associated with dysbiosis and weakening of the gut barrier integrity, exposing the intestinal immune system to various microbial pathogens [258, 259], some of which may exhibit molecular mimicry of certain autoantigens of the host. For instance, commensal bacteria expressing orthologs of Ro60, an RNA-binding protein commonly targeted by autoAbs in SLE, can trigger autoimmunity in genetically susceptible individuals [260]. In ulcerative colitis and some CD patients, anti-neutrophil cytoplasmic antibodies (ANCA) are strongly correlated with neutrophil extracellular traps (NET) formation and increased gut permeability [261–264]. ANCA also exhibits cross-reactivity with the outer membrane porin C (OmpC) of *Bacteroides caccae* and *Escherichia coli*, further instigating the inflammatory bowel disease (IBD) [265]. Additional self-antigens and microbial antigens exhibiting cross-reactivity with self-antigens, including glycoprotein 2 (GP2) and family with sequence similarity 84 member A (FAM84A), have been reviewed [266].

In addition, increased gut permeability during aging and obesity also allows for increased exposure of pathogenic bacteria and LPS to GC B cells in the intestinal TLS, accelerating B cell inflammatory function, senescence-like changes, leading to less IgA beneficial for homeostasis, decreased IgA diversity, and increased IgA autoAbs [261, 267–269], further contributing to inflammaging and immunosenescence [263] (Fig. 1B). Future research will need to elucidate the relationship between autoAbs and the microbiome in the context of aging and obesity. Moreover, it is also worth studying how B cells traffic from the intestine can impact tissue-specific inflammation and autoAb production across other tissues, or how gut-related therapies, including microbial mimics and dietary interventions could be used to target gut inflammation and/or pathogenic antibody production during obesity or aging to curb these conditions [264, 270]. Altogether, gut-derived antibodies contribute to the persistence of inflammation by continuously stimulating the intestinal immune system, leading to tissue damage and chronic inflammation associated with immunosenescence, impacting the gut’s ability to respond to constant bacterial challenges and maintain immune tolerance.

### Central nervous system

Anti-neuronal autoAbs have been found in the periphery and in several brain-associated regions including the cortex [271], in the meningeal dura mater [272], and cerebrospinal fluid [273]. Here, they are expected to drive pathology in cases of autoimmune disease. In age-related cognitive conditions like PD and AD, autoAbs can drive both protective and deleterious effects (Fig. 1B). AutoAbs and other antibodies have been best studied in AD. Anti-amyloid antibodies are found at differential levels in AD patients, partially representing the sequestration by plaques [274]. These autoAbs are expected to be protective, targeting plaques for clearance by the abundant microglia population in AD, even being developed into drug candidates [275]. Given the linkages between APOE and AD, antibodies against APOE isoforms are being developed [276] but there is little evidence of their natural occurrence and autoAbs in AD. AutoAbs against other amyloid family proteins also represent an interesting AD resilience factor. The pancreas-derived islet amyloid polypeptide (IAPP)-IgA autoAb was also significantly lower in APOE4 carriers [277]. The IgA isotype is generated in the mucosa upon sampling of antigens encountered in tissues like the gut and lung [278]. Conversely, several other autoAbs were found at higher levels that were not neuron-specific [279], also representing chronic inflammation. Features of insulin resistance have been linked to AD and neurodegeneration [280]. The protective IgA class of antibodies generated in the gut is reduced in obese mice [262]. Whether this leads to increased levels of protective autoAbs or specifically neuroinflammation is unclear. As mentioned above, GFAP may be another CNS target incited during obesity, and studies in rats have shown increased deposition of IgG in the brains of HFD rodents, correlating with infiltration of microglia [184].

In PD, autoAbs against alpha-synuclein were found in increasing quantities in blood and cerebrospinal fluid (CSF) of patients, with a bias in males [281]. These synuclein autoAbs are expected to target neurons, drive inflammation and promote PD pathology [282]. Also, GFAP autoAbs found in DIO target cells like astrocytes and expected to correlate with astrogliosis and PD [274]. Interestingly, renin-angiotensin system (RAS) autoantibodies were also increased in PD serum [283], likely representing systemic inflammation linked age-related breakdown in tolerance.

## Potential therapeutics extending lifespan and healthspan

**Fig. 2:**
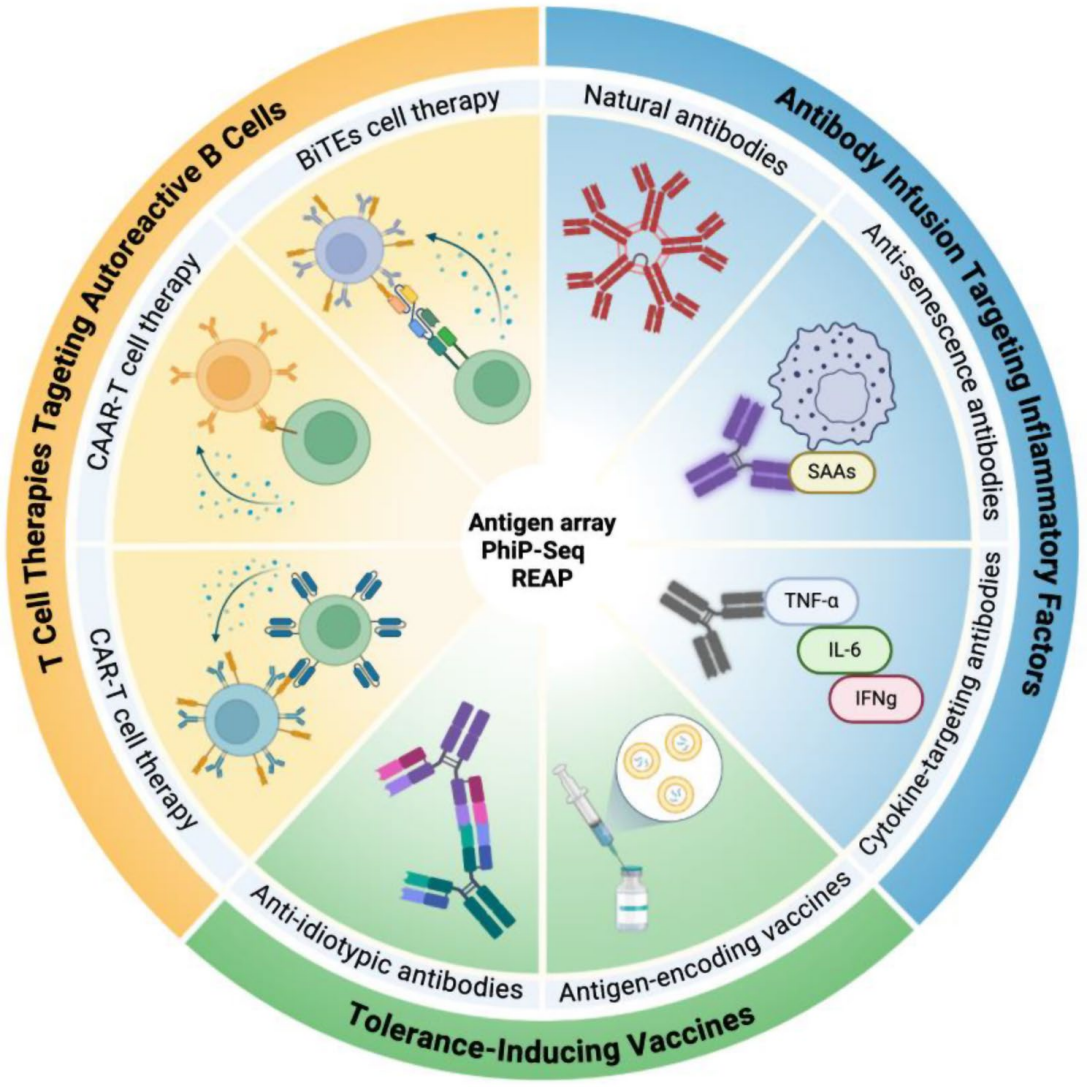
Potential therapies targeting autoimmune components during aging and obesity. With next-generation autoAb detection methods, such as PhiP-Seq, antigen arrays, and REAP, playing a central role in providing a more detailed atlas of autoAbs, therapies traditionally used for autoimmune diseases can be repurposed to conquer the autoAbs and autoantigens generated during obesity and aging. T cell-based therapies targeting autoreactive B cells, antibody-based therapies targeting pro-inflammatory factors, and tolerance-inducing vaccines based on anti-idiotypic antibodies and antigen-encoding mRNA vaccines, are emerging techniques that are gaining increasing research focus. PhiP-Seq = phage immunoprecipitation sequencing; REAP = rapid extracellular antigen profiling; CAR-T = Chimeric antigen receptor T cells; CAAR-T = Chimeric autoantibody receptor T cells; BiTEs = Bispecific T cell engagers; SAAs = senescence-associated antigens

Given the critical role that autoAbs play in driving chronic inflammation during aging and obesity, the potential of therapies traditionally purposed for autoimmune diseases in resolving inflammaging and improving health/lifespan is worth exploring. However, although autoAbs can have significant clinical effects and provide valuable insights for drug development, the employment of therapies targeting autoAbs or autoreactive B cells to counteract inflammaging or obesity still represents an understudied niche. A major obstacle has been the absence of experimental tools capable of unbiased, high-throughput detection of autoAbs across the human population on a proteome-wide scale. However, technologies, such as rapid extracellular antigen profiling (REAP), antigenic arrays, and phage immunoprecipitation sequencing (PhIP-seq) [284, 285], will allow for “autoAb-wide association studies”, enabling the identification of disease-modifying autoAbs not only for autoimmune diseases but also for aging and metabolic diseases [13] (Fig. 2). Consistently, one study mapping over 730,000 human-derived peptides showed that each individual, regardless of disease states, has unique and complex finger-prints of autoAbs that were best targeted using therapies against plasma cell markers like B cell maturation antigen (BCMA), instead of anti-CD19 or anti-CD20 [286].

However, recent advancements in treating autoimmune diseases have been made by targeting autoAb-secreting B cells, an integral component to be reset during the sequential immunotherapy of autoimmune diseases [287]. Specifically, chimeric antigen receptor (CAR) T-cells targeting CD19 have been shown to successfully treat a patient with severe myositis and systemic sclerosis [288], as well as severe, anti-acetylcholine receptor antibody (anti-AchR) positive generalized MG [289]. B cells producing specific autoAbs can also be targeted by the chimeric autoantibody receptor (CAAR) T cells, which are engineered to produce and present specific antigens on their surface, allowing for recognition by corresponding B cells, which are consequently eliminated. Successful application of CAAR T cells has been made in treating anti-NMDA receptor (NMDAR) autoAb-mediated NMDAR encephalitis, the most common autoimmune encephalitis [290], as well as in anti-desmoglein 3 autoAb-mediated pemphigus vulgaris [291]. The CAR-based therapies have also shown the potential in aging research, for example, by targeting senescent cells markers, like urokinase plasminogen activator receptor (uPAR), to ameliorate the metabolic function decline in aged mice or DIO mice [292, 293]. Meanwhile, various senolytics or clearance of senescent cells, such as p21^high^ cells, have shown efficacies in improving physiological functions during aging [294–296]. This research implicates the possibility of targeting senescent cells with antibodies against senescence-associated peptides, such as those encoded by *Tns3* and *Tmed3* in mice, which have shown high immunogenicity [297]. However, CAR-based therapies targeting age-related autoAbs warrant further investigation. Remarkably, with the newly developed CAR-enhancer technology and other CAR therapy improvements, the therapeutic potency of canonical CAR-based therapies will be significantly amplified [298, 299]. As well, additional approaches, such as the bispecific T cell engagers (BiTE) (e.g. blinatumomab that targets autoreactive B cells in RA) [300], may further expand the future therapeutic options for ARDs.

Importantly, some autoAbs possess dualistic properties that are sometimes beneficial and are less appreciated. For instance, IgG autoAbs generated in the WAT of DIO mice are found to facilitate phagocytosis of apoptotic adipocytes by macrophages, suggesting a beneficial role by some autoAbs during obesity [301]. Additional examples include anti-IFN-I autoAbs that can reduce SLE severity [302], anti-human epidermal growth factor receptor 2 (HER2) autoAbs that increase breast cancer patients’ survival rate [303], and anti-amyloidogenic peptides autoAbs that protect against AD [304]. Monoclonoal antibody-based drugs have been developed based on the antibodies above, including Anifrolumab for SLE [305], Trastuzumab for breast cancer [306], and Aducanumab for AD [307], indicating that autoAbs exhibit promising therapeutic value depending on different pathological conditions.

In addition, vaccines targeting autoAbs and “tolerance-inducing vaccines” targeting autoantigens have been gaining focus recently [308]. To avoid global immunosuppression during the induction of immune tolerance, antigen-specific tolerance represents a future avenue. For example, conjugating the antigen with a polymer glycosylated with N-acetylgalactosamine (pGal) induced antigen-specific tolerance mediated by Tregs in mice with experimental autoimmune encephalomyelitis (EAE) [309]. More importantly, anti-idiotypic antibodies, which target the idiotype of other antibodies, have gained interest for their potential to counteract harmful autoAbs in autoimmune diseases [310]. These antibodies are integral to the immune system’s regulation, as they can neutralize and inhibit the secretion of autoAbs, offering a novel form of immunotherapy with minimal side effects and prolonged immunity [311, 312]. While their application in age-related and metabolic diseases is not yet well-studied, emerging techniques like antigenic arrays, REAP, and PhIP-Seq are expected to identify candidate autoAbs, paving the way for vaccines targeting autoABs in these conditions.

## Summary and concluding remarks

Aging and obesity contribute to autoAb production through chronic inflammation, immune dysregulation, and metabolic disturbances [7, 19]. Inflammaging is associated with disrupted immune tolerance and increased abundance of ABCs and other autoreactive B cells, leading to the production of autoAbs, such as anti-dsDNA and ANA IgG linked to AD and cardiovascular diseases [108, 189]. Obesity promotes similar effects through metainflammation, activating B cells through metabolic reprogramming towards a hypermetabolic, autoAb-secreting state [84]. Structural changes in antibodies, such as altered glycosylation, increase self-reactivity [162], while autoantigens can be generated through PTMs and metabolic disturbances, like AGE-modified proteins and glycosphingolipids, further exacerbating autoimmune responses [186].

**Fig. 3:**
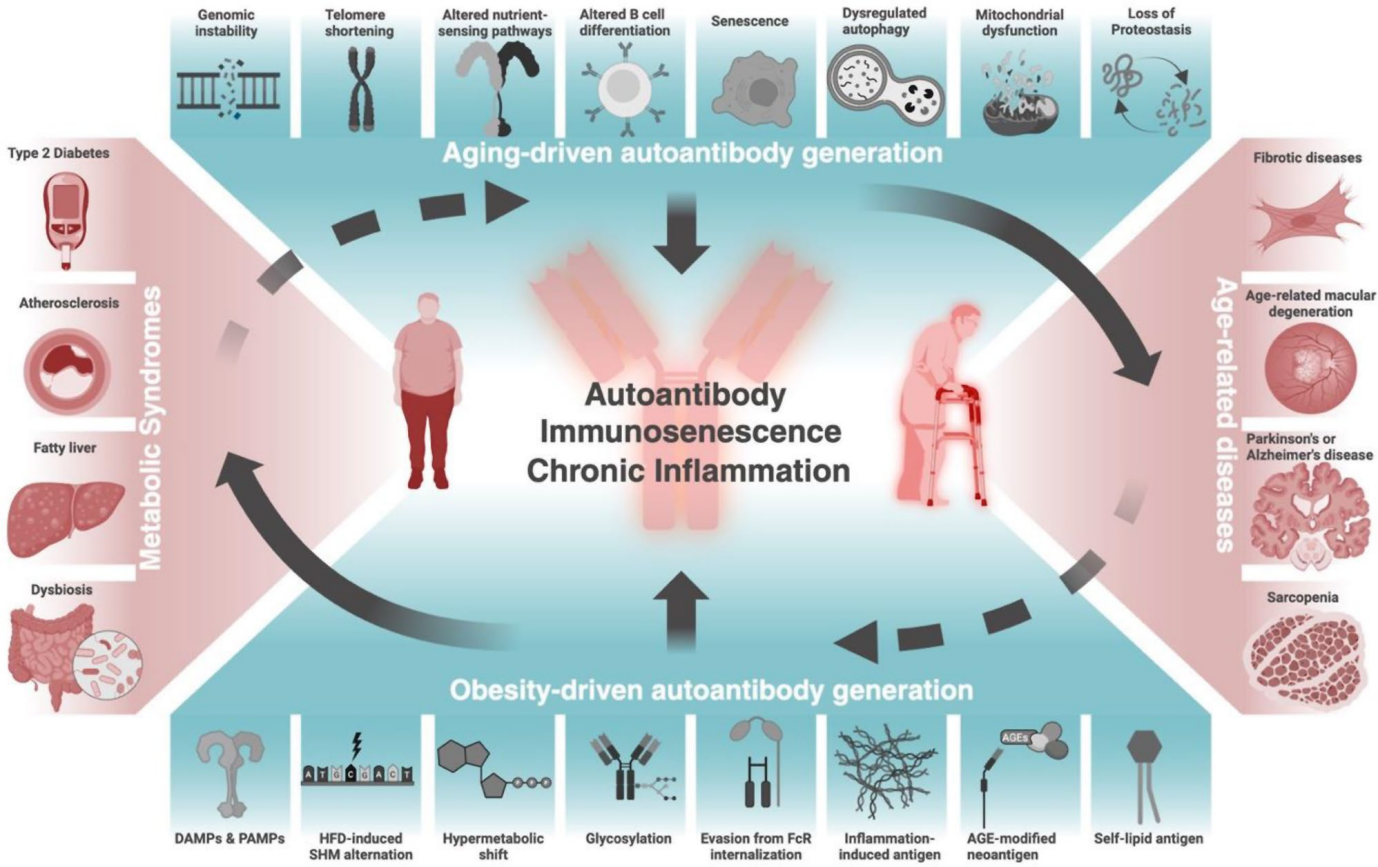
A vicious cycle of immune dysregulation during obesity and aging mediated by autoantibodies. During obesity and aging, the generation of autoantibodies depends on various interconnecting mechanisms, including ones that directly contribute to alteration in B cell functions and antibody structures, as well as several hallmarks of aging that promote the formation of autoantigens. The autoantibodies then play a critical role in fueling chronic inflammation that eventually contributes to immunosenescence, which continues to drive age-related diseases and metabolic syndromes. While obesity-related autoantibodies can contribute to metabolic syndromes, and age-related autoantibodies can contribute to age-related diseases (denoted as solid arrows), metabolic syndromes and age-related diseases can potentially create niches for the generation of age-related and obesity-related autoantibodies, respectively (denoted as dashed arrows). As a result, a vicious cycle between metabolic syndromes and age-related diseases forms and can exacerbate each other when driven by immunosenescence and chronic inflammation associated with autoantibodies. AGE = Advanced glycation end product; DAMPs = Danger-associated molecular patterns; PAMPs = Pathogen-associated molecular patterns; HFD = High-fat diet; SHM = Somatic hypermutation

These changes to antibodies during obesity and aging occur in the setting of chronic low-grade inflammation. Chronic inflammation is an important hallmark of aging, not only as a pathological consequence of disturbed homeostasis during aging but for its ability to instigate further age-related dysfunction [5]. AutoAbs resulting from obesity and aging can drive a common feedforward loop that promotes metabolic diseases, immune-mediated conditions, and a decline in immune function [147] (Fig. 3). In metabolic tissues, the gut, and the CNS, autoAbs lead to sustained immune activation, tissue damage, and oxidative stress, perpetuating a cycle of immune dysregulation [313, 314]. The chronic state of inflammation accelerates the aging of the immune system, resulting in immunosenescence, which in turn can promote a positive feedback loop that drives metabolic diseases and impaired health.

The role of autoAbs in obesity, aging, and immunosenescence is a complex, yet vital, research area. Fueled by chronic inflammation, autoAbs not only accelerate the progression of metabolic syndrome and ARDs but also perpetuate a cycle of immune dysregulation that drives immunosenescence and impairs the healthspan. The persistent immune activation and tissue damage across various organs underscore the extensive impact of autoAbs on ARD progression. The rise in autoimmune diseases among the elderly, despite declining immune competence, is caused by a breakdown of immune tolerance. The accumulation of age-related failures in both central and peripheral tolerance likely contributes to the emergence of autoimmunity. Yet, significant gaps in our knowledge persist, including the extent to which these defects are intrinsic to B cells and how they interact with the systemic immune system.

A primary challenge is deciphering the molecular components and signaling networks that lead to tolerance breaches and subsequent autoAb generation. The heterogeneity and temporal variability of autoAbs present a substantial barrier, complicating our understanding and necessitating large cohort studies and longitudinal analyses [13]. In addition, while the role of autoAbs is central, the influence of genetic and environmental factors, as well as lifestyle needs to be considered [110]. To address the age-related generation of autoAbs, future research must investigate the cellular and molecular mechanisms by which immune aging transforms immune cells into auto-aggressive effectors. This includes investigating mitochondrial dysfunction, lysosomal failure, and chronic ER stress and expansion in T cells and B cells, as well as their communication with the environment, including microbes and the environmental exposome [101]. In addition, given the PDIA3 enrichment in macrophages in tissues of altered stiffness due to obesity and chronic inflammation, changes in potentially mechanosensing self-antigen levels might represent a novel target of study to ameliorate aging and obesity-related conditions [210]. In conclusion, by defining the interplay among various risk factors, we can enhance our understanding of the intrinsic and extrinsic factors that shape immunity as we age, paving the way for promising therapeutic strategies to combat autoimmune conditions associated with obesity and ARDs.

## Data Availability

No datasets were generated or analysed during the current study.

## Abbreviations

APRIL: A prolifeting-inducing ligang
AchR: Acetylcholine receptor
AID: Activation-induced cytidine deaminase
AGEs: Advanced glycated end products
ABC: Age-associated B cell
ARDs: Age-related diseases
AMD: Aged-related macular degeneration
ALDH4A1: Aldehyde dehydrogenase 4 family member A1
AD: Alzheimer’s disease
ACPA: Anti-citrullinated protein antibodies
ADCC: Antibody-dependent cellular cytotoxicity
ADE: Antibody-dependent enhancement
ASCs: Antibody-secreting cells
ANCA: Anti-neutrophil cytoplasm antibody
AAV: Anti-neutrophil cytoplasm antibody-associated vasculitis
ANA: Antinuclear antibody
E-APOE: Apolipoprotein
AIM: Apoptosis inhibitor of Macrophages
AutoAb: Autoantibodies
BAFF: B cell activating factor
BAFF-R: B cell activating factor receptor
BLIMP-1: B lymphocyte-induced maturation protein 1
BCR: B cell receptor
BiTE: Bispecific T cell engager
BM: Bone marrow
BAT: Brown adipose tissue
CVD: Cardiovascular disease
CNS: Central nervous system
CSF: Cerebrospinal fluid
CAR: Chimeric Antigen Receptor
CAAR: Chimeric Autoantibody Receptor
CSR: Class switch recombination
COVID-19: Coronavirus disease 2019
CD: Crohn’s disease
CXCL12: C-X-C Motif Chemokine Ligand 12
CXCR4: C-X-C chemokine receptor type 4
DAMPs: Danger-associated molecular patterns
DNA: Deoxyribonucleic acid
DCs: Dendritic cells
DIO: Diet induced obesity
DNA-PKcs: DNA-dependent protein kinase
DN2: Double negative
dsDNA: Double stranded DNA
DMD: Dystrophin
eWAT: epididymal white adipose tissue
EAE: Experimental autoimmune encephalomyelitis
eIF3: Eukaryotic translation initiation elongation factor-3
FDC: Follicular dendritic cell
Tfh: Follicular helper T cells
FcR: Fragment crystallizable receptor
FcRn: Fragment crystallizable receptor neonatal
GAPDH: Glyceraldehyde 3-phosphate dehydrogenase
GC: Germinal Center
GFAP: Glial fibrillary acidic protein
GRP78: Glucose regulated protein 78
GSL: Glyosphingolipid
GP2: Glycoprotein 2
HSP: Heat shock protein
HFD: High fat diet
HFHF: High Fructose/High Fat Diet
HRS/Jo-1: Histidyl tRNA synthetase
HER2: Human epidermal growth factor receptor 2
HIV: Human immunodeficiency virus
IFN: Interferon
IL: Interleukin
Ig: Immunoglobulin
ITAM: Immunoreceptor tyrosine-based activation motif
ITIM: Immunoreceptor tyrosine-based inhibitory motif
IRGM1: Immunity-related GTPase family M member 1
iNOS: Inducible nitric oxide synthase
IGF: Insulin-like growth factor
iNKT: Invariant natural killer T cell
IAPP: Islet amyloid polypeptide
KO: Knock out
LPS: Lipopolysaccharide
Ldlr: Low density lipoprotein receptor
MDA: Malondialdehyde
MetS: Metabolic syndrome
mtDNA: Mitochondrial DNA
mtRNA: Mitochondrial RNA
mTOR: Mammalian target of rapamycin
MYH: Myosin heavy chain
MALT: Mucosa-associated lymphoid structure
MS: Multiple Sclerosis
Mtb: Mycobacterium tuberculosis
GlcNAc: N-acetylglucosamine
NK cells: Natural killer cells
NMDAR: N-methyl-D-aspartate receptor
NLR: Nod like receptors
NET: Neutrophil extracellular traps
OmpC: Outer membrane porin C
OXPHOS: Oxidative Phosphorylation
PD: Parkinson’s disease
PI3Kγ: Phosphoinositide 3-kinase-gamma
PhIP-seq: Phage immunoprecipitation sequencing
pGal: Polymer glycosylated with N-acetylgalactosamine
PTM: Post-translational modification
REAP: Rapid extracellular antigen profiling
Bregs: Regulatory B cells
Tregs: Regulatory T cells
RAS: Renin-angiotensin system
RA: Rheumatoid Arthritis
RNA: Ribonucleic acid
RPS6KA6: Ribosomal protein S6 kinase
SAAs: Senescence-associated antigens
SARS-CoV-2: Severe acute respiratory syndrome coronavirus 2
SHM: Somatic hypermutation
SLO: Secondary lymphoid organs
SASP: Senescence-associated secretory phenotype
SS: Sjögren’s syndrome
SAT: Subcutaneous adipose tissue
SLE: Systemic lupus erythematosus
TERF: Telomeric repeat-binding factor
TLS: Tertiary lymphoid structures
TLR: Toll-like receptor
TGF: β-Transforming growth factor beta
TNF: Tumor necrosis factor
T2D: Type 2 diabetes
VAT: Visceral adipose tissue
WT: Wild Type
